# Interactions of Microbiota and Mucosal Immunity in the Ceca of Broiler Chickens Infected with *Eimeria tenella*

**DOI:** 10.3390/vaccines10111941

**Published:** 2022-11-17

**Authors:** Janghan Choi, Wookyun Kim

**Affiliations:** Department of Poultry Science, University of Georgia, Athens, GA 30602, USA

**Keywords:** *Eimeria tenella*, broilers, microbiome, proteobacteria, firmicutes, mucosal immunity, goblet cells

## Abstract

The purpose of the study was to investigate the effects of *Eimeria tenella* infection on the cecal microbiome, the protein concentration of cecal content, cecal mucosal immunity, and serum endotoxin levels in broilers. Three hundred sixty 14-day-old broilers were allocated to five infection doses with six replicates. The five infection doses were: ID0: 0, ID1: 6250, ID2: 12,500, ID3: 25,000, and ID4: 50,000 *Eimeria tenella* oocysts. *Eimeria tenella* infection significantly increased the relative abundance of the phylum Proteobacteria, which includes diverse pathogenic bacteria, and significantly decreased the relative abundance of the phylum Firmicutes. Protein concentration of the cecal content was linearly increased (*p* < 0.05), and the concentration of secretory immunoglobulin A (sIgA) in the cecal content was linearly decreased by *Eimeria tenella* infection (*p* < 0.05). Goblet cell density was linearly reduced in the ceca by *Eimeria tenella* infection (*p* < 0.05). *Eimeria tenella* infection tended to linearly decrease the relative mRNA expression of antimicrobial peptide genes such as avian beta-defensin 9 (AvBD9; *p* = 0.10) and liver-expressed antimicrobial peptide 2 (LEAP2; *p* = 0.08) in the cecal tissue. Therefore, *Eimeria tenella* infection negatively modulated cecal microbiota via impairing cecal mucosal immunity and increasing protein concentration in the cecal content in broilers.

## 1. Introduction

Coccidiosis, an enteric disease caused by the genus *Eimeria*, results in severe economic loss higher than USD 10 billion annually in the world due to reduced production efficiency and increased treatment costs [1,2,3]. There are seven *Eimeria* spp. that can infect chickens, and each *Eimeria* spp. colonizes different parts of gastrointestinal tracts and exhibits negative impacts on chickens with different modes of actions [4]. *Eimeria tenella* resides in the epithelial cells of cecal crypts in broilers and modulates cecal functionality [5]. *Eimeria tenella* infection is known to reduce growth performance, mainly feed efficiency [6], induce oxidative stress [7], induce inflammatory response [8], and modulate gut barrier integrity [9] in broilers. *Eimeria tenella* is known to be one of the most pathogenic *Eimeria* spp. that causes high morbidity and mortality in broiler chickens [10].

The negative effects of *Eimeria tenella* infection in broilers are closely associated with modulated cecal microbiota, and several studies have shown that *Eimeria tenella* infection can negatively affect cecal microbiota in broilers [11,12,13]. Cecum is the main area for microbial fermentation and is a reservoir for diverse commensal and pathogenic bacteria in chickens [14]. The cecal microbiota plays an important role in producing volatile fatty acids (VFA), important energy sources for the host, by degrading cellulose or other host-indigestible carbohydrates [15]. The ceca also harbor pathogens including *Salmonella* spp., *Escherichia coli*, and *Shigella* spp. that can cause growth retardation and inflammation by increasing serum endotoxin levels in chickens [16].

The cecal microbiome can be modulated due to diverse factors including pathogenic infection [17], nutrient composition of digesta content [18], oxidative stress [19], and immune system [20]. However, the relationship between *Eimeria tenella* infection and the microbiome of broilers and the effects of modulated microbiota on serum endotoxin level are still unclear, and it is essential to demonstrate their interactions to effectively control the negative effects of *Eimeria tenella* infection in broilers. We hypothesized that modulated nutrient composition of cecal content and mucosal immunity due to *Eimeria tenella* infection negatively affects cecal microbiome, leading to an increase in serum endotoxin levels in broiler chickens. This study was designed to research the interactions of cecal microbiome with protein concentration of cecal content, cecal mucosal immunity, and serum endotoxin levels in broilers infected with different infection doses of *Eimeria tenella*.

## 2. Materials and Methods

### 2.1. Experimental Design and Sampling

The study was conducted at the Poultry Research Center, University of Georgia, Ath-ens, GA, USA and approved by the Institutional Animal Care and Use committee of the University of Georgia (A2018 09-006). Three hundred sixty 14-day-old male Cobb 500 broilers were divided to 5 infection doses with 6 replicates of 12 birds per cage in a completely randomized design. The different infection doses were (1) infection dose 0 (ID0): administration with 1 mL of phosphate-buffered solution (PBS) as a sham-challenged group; (2) infection dose 1 (ID1): administration with 6250 sporulated oocysts of *Eimeria tenella*; infection dose 2 (ID2): administration with 12,500 sporulated oocysts of *E. tenella*; infection dose (ID3): administration with 25,000 sporulated oocysts of *E. tenella*; and infection dose (ID4), administration with 50,000 sporulated oocysts of *E. tenella*. The administration was conducted via oral gavage of 1 mL PBS or *Eimeria tenella* inoculum. The *Eimeria tenella*, a wild-type strain, was freshly isolated and prepared (within 3 months). Broilers were fed the experimental diet formulated according to the 2018 Cobb Broiler Nutrition management to meet or exceed the requirement level of the specific age (Table 1). Birds had free access to water and feed during the whole experimental period and were reared according to the recommendation of 2018 Cobb Broiler Management Guide.

On 6 days post-infection (dpi), one bird per pen (e.g., an experimental unit) was euthanized via cervical dislocation. Afterward, blood from the heart was collected into heparin-free vacutainer tubes (Grainer Bio-One, Kremsmuenster, Austria). The collected blood samples stood at room temperature for 1 h for clotting and were centrifuged at 1000× *g* for 15 min to recover the serum. Mid-cecal contents and tissues were collected and snap-frozen in liquid nitrogen. Serum and snap-frozen samples were kept at −80 °C for further analyses. Around 3 cm of the mid-ceca section was collected and fixed in 10% neutral-buffered formalin.

### 2.2. DNA Extraction and Microbiome Analyses in the Cecal Content

DNA was extracted from approximately 100 mg of the contents of the mid-ceca using QIAamp^®^ DNA stool mini kits (Qiagen GmbH, Hilden, Germany) according to the manufacturer’s procedure. Quality and quantity of extracted DNA were checked utilizing a NanoDrop 2000 spectrophotometer (Thermo Fisher Scientific, Waltham, MA, USA). The 16s rRNA gene sequencing was performed by LC sciences, LLC (Houston, TX, USA) according to Choi et al. [21]. The amplification of the V3 and V4 regions was conducted using 338F (5-CCTACGGGNGGCWGCAG-3)/806R (5-GACTACHVGGGTATCTAATCC-3) primers by PCR procedures. QIIME2 (version 2022.02) was utilized for 16s rRNA analyses in the cecal microbial communities [22]. QIIME2 plugin DADA2 was used to denoised demultiplexed sequence. For taxonomical classification, GreenGenes database (version 13.8) at the 99% operational taxonomic units (OTUs) was used [21]. Phylum- and family-level compositions with the relative abundance of genus Shigella were shown. By QIIME2′s built in functions, the parameters of alpha diversity including shannon entropy (richness and evenness), observed features (richness), faith phylogenetic diversity (biodiversity based on phylogeny), pielou evenness (evenness), and beta diversity (bray curtis, jaccard, and unweighted and weight unifrac) of cecal microbial communities were determined.

### 2.3. Determination of Secretory Immunoglobulin A (sIgA) and Protein Concentration in the Cecal Contents

Approximately 100 mg cecal content was homogenized in 1 mL of PBS with 0.1 mm beads for 45 s using a beads beater (Biospec Products, Bartlesville, OK, USA). The concentration of secretory immunoglobulin A (sIgA) in the cecal content was determined by an IgA Chicken ELISA test kit (Abcam, Cambridge, UK) after 2000 times sample dilution according to the manufacture’s protocol. The protein concentration was analyzed by using the Pierce™ BCA Protein Assay Kit (Thermo Fisher Scientific, Cleveland, OH, USA) after 20 times dilution according to the manufacture’s protocol. The absorbance was determined using SpectraMax^®^ ABS Plus microplate reader (Molecular devices, San Jose, CA, USA). The concentrations of sIgA and protein were expressed as values per g sample.

### 2.4. RNA Extraction and Real-Time Reverse Transcription (RT)-PCR Analysis for Analyzing Relative mRNA Abundance of Antimicrobial Peptides, Alkaline Phosphatase, Mucin Gene, Inflammatory Cytokines, and Toll-Like Receptors

Around 100 mg of the mid-ceca tissue samples were homogenized in QIAzol lysis reagents (Qiagen, Valencia, CA, USA) using a bead beater (Biospec Products), and RNA was extracted according to the manufacturer’s procedure. RNA quantity and purity were checked using a NanoDrop 2000 spectrophotometer (Thermo Fisher Scientific). The first-strand cDNA was synthesized by using one μg RNA with high-capacity cDNA synthesis kits (Applied Biosystems, Foster City, CA, USA). Table 2 lists the primers used in the study. Real-time reverse transcription (RT) PCR was conducted by utilizing SYBR Green Master Mix with a Step One thermocycler (Applied Biosystem). The final PCR volume (10 μL) contained 5 μL of SYBR Green Master Mix, 1.5 μL of cDNA, 0.5 μL of forward and reverse primers (10 μM), and 2.5 μL of water. Thermal cycle conditions for all reactions were 95 °C denatured for 10 min, 40 cycles at 95 °C for 15 s and 60 °C for 1 min, and 95 °C for 15 s, 60 °C for 1 min, and 95 °C for 15 s. The housekeeping genes (reference genes) were Glyceraldehyde 3-phosphate dehydrogenase (GAPDH) and beta-actin, and the mRNA abundance of the target genes was normalized by using geometric means of housekeeping genes [23]. Relative mRNA abundance of target genes was determined using the 2^−∆∆Ct^ method [24].

### 2.5. Quantification of Goblet Cells by Using Alcian Blue/Period Acid-Schiff (AB/PAS) Staining

To enumerate goblet cell per crypt and per 100 μm crypt depth (CD) in the mid-ceca, alcian blue/period acid-schiff (AB/PAS) staining was utilized [25]. The mid-cecal samples were cut by blades and put in cassettes. Each section was stained with alcian blue for 15 min and washed using distilled water. Samples were treated with periodic acid for 5 min and washed with distilled water. Afterwards, the samples were stained with Schiff’s reagents for 10 min and washed with distilled water. They were counterstained in hematoxylin for 1 min, washed, and dehydrated. The stained sections were viewed with a BZ microscope (BZ-X810; Keyence, Osaka, Japan). The captured images (4×) were analyzed using Image J (National Institutes of Health, Bethesda, MD, USA).

### 2.6. Alkaline Phosphatase Activities in the Cecal Tissue and Serum and Serum Endotoxin Levels

Alkaline phosphatase activities in the cecal tissue and serum were determined according to Lackeyram et al. [26] with some modifications. For cecal tissue, approximately 100 mg of tissue samples was homogenized with the one milliliter of homogenization buffer (pH 7.4) containing 50 mM D-mannitol, 10 mM Trizma.HCl, and 10 mM Hepes. For the analyses of protein content using Pierce™ BCA protein assay kits (Thermo Fisher Scientific), the supernatants were used after 10 times dilution. Cecal tissue samples were diluted 2 times, and serum samples were diluted 10 times with the pre-substrate solution containing 2.08 mM potassium fluoride, 50 mM NaHCO_3_, 50 mM Na_2_Co_3_, 5 mM MgCl_2_.6H_2_O, 10 mM Trizma.HCl, and 10 mM Hepes. The 180 μL of the substrate solution containing 20 mM p-nitrophenyl phosphate was added to each well containing 20 μL of sample solution in a 96 well plate. The plate was incubated for 30 min at 37 °C. The absorbance of 400 nm was determined using a SpectraMax^®^ ABS Plus microplate reader (Molecular devices, San Jose, CA, USA). The concentration of the enzyme product (p-nitrophenol) was used to represent alkaline phosphatase activities and was quantified by a prepared standard curve. Alkaline phosphatase activities in the cecal tissue were normalized with the protein concentration of the solution. Alkaline phosphatase activities in the cecal tissue were expressed as p-nitrophenol concentration per serum mL.

Serum endotoxin concentration was determined using a Pierce™ LAL Chromogenic Endotoxin Quantitation Kit (Thermo Fisher Scientific) after 10 times dilution in endotoxin-free water.

### 2.7. Statistical Analyses

Statistical analyses were conducted using SAS (version 9.4; SAS Inst. Inc., Cary, NC, USA). All groups were compared using PROC MIXED (ANOVA) in a completely randomized design followed by the Tukey’s comparison test. Orthogonal polynomial contrasts were performed to evaluate the significance of the linear or quadratic effects of different *Eimeria tenella* infection doses, and the infection doses of *E. tenella* were normalized by using the base 2 logarithm of the number of sporulated *E. tenella* number for orthogonal polynomial contrasts [6]. Statistical significance was set at *p* < 0.05, and trends (0.05 ≤ *p* ≤ 0.1) were also presented.

## 3. Results

### 3.1. Alpha and Beta Diversity of the Cecal Microbial Communities

**Figure 1:**
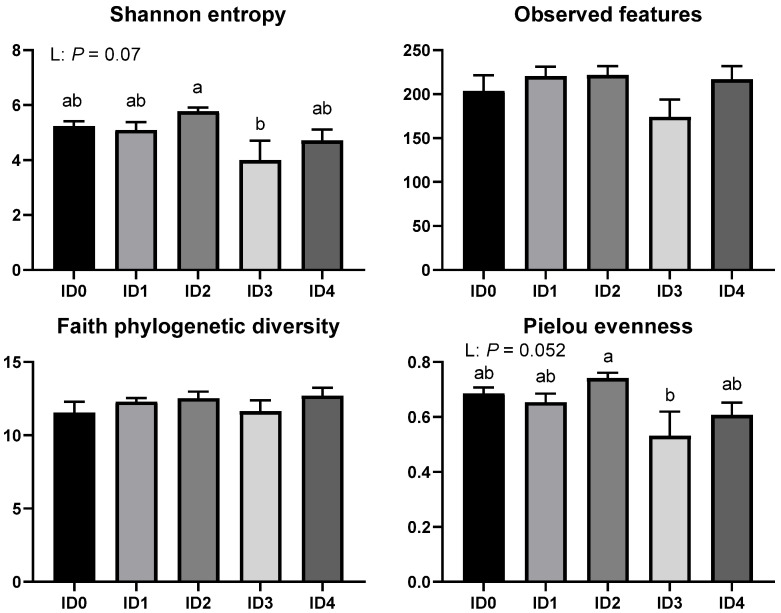
Effects of different infection doses of *Eimeria tenella* on alpha diversity parameters of the cecal microbial communities in broilers 6 days post-infection. Alpha diversity parameters were measured in the ID0 (infection dose 0; Sham-challenged with phosphate-buffered saline); ID1 (infection dose 1; challenged with 6250 sporulated oocysts of *Eimeria tenella*); ID2 (infection dose 2; challenged with 12,500 sporulated oocysts of *E. tenella*); ID3 (infection dose 3; challenged with 25,000 sporulated oocysts of *E. tenella*); ID4 (infection dose 4; challenged with 50,000 sporulated oocysts of *E. tenella*) groups. Data were analyzed by using One-way ANOVA followed by the Tukey’s multiple comparison test and means with different letters (a and b) differ significantly (*p* < 0.05). Orthogonal polynomial contrast analysis was used to assess linear (L) and quadratic (Q) effects of the infection doses, and *p* < 0.1 were presented.

The alpha diversity parameters (biodiversity of the samples) including shannon entropy (richness and evenness), observed features (richness), faith phylogenetic diversity (biodiversity based on phylogeny), and pielou evenness (evenness) are shown in Figure 1. *Eimeria tenella* infection tended to decrease alpha diversity parameters including shannon entrophy (*p* = 0.07) and pielou evenness (*p* = 0.052) in the cecal microbial communities. The ID2 group had significantly greater shannon entropy (richness and evenness) and pielou evenness compared to the ID3 group (*p* < 0.05).

**Figure 2:**
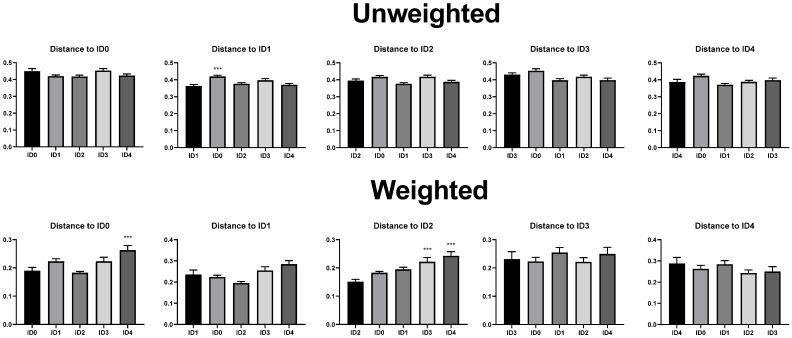
Effects of different infection doses of *Eimeria tenella* on beta diversity parameters (unweighted and weighted unifrac) of the cecal microbial communities in broilers on 6 days post-infection. Beta diversity parameters were measured in the ID0 (infection dose 0; sham-challenged with phosphate-buffered saline); ID1 (infection dose 1; challenged with 6250 sporulated oocysts of *E. tenella*); ID2 (infection dose 2; challenged with 12,500 sporulated oocysts of *Eimeria tenella*); ID3 (infection dose 3; challenged with 25,000 sporulated oocysts of *Eimeria tenella*); ID4 (infection dose 4; challenged with 50,000 sporulated oocysts of *Eimeria tenella*) groups. The infection dose groups were compared using one-way ANOVA followed by Dunnett’s post hoc test. Each infection dose group was set as the control group. *** presented when *p* < 0.01. Orthogonal polynomial contrast analysis was used to assess linear (L) and quadratic (Q) effects of the infection doses, and *p* < 0.1 were presented.

**Figure 3:**
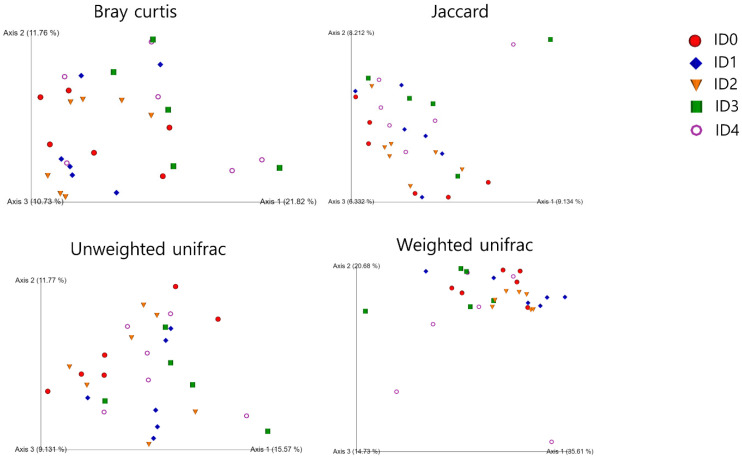
Visualized beta diversity parameters including bray curtis, jaccard, and weighted and unweighted unifrac in the *Eimeria tenella* infected broiler groups 6 days post-infection: ID0 (infection dose 0; sham-challenged with phosphate-buffered saline); ID1 (infection dose 1; challenged with 6250 sporulated oocysts of *Eimeria tenella*); ID2 (infection dose 2; challenged with 12,500 sporulated oocysts of *E. tenella*); ID3 (infection dose 3; challenged with 25,000 sporulated oocysts of *E. tenella*); ID4 (infection dose 4; challenged with 50,000 sporulated oocysts of *Eimeria tenella*).

Distance to each infection dose in the unweighted unifrac beta diversity (dissimilarity among samples without considering abundance information) analysis is shown in Figure 2. The ID0 group had significantly greater unweighted unifrac distance to the ID1 group compared to the ID1 group. The ID4 group had significantly greater weighted unifrac distance (dissimilarity among samples with considering abundance information) to the ID0 group compared to the ID0 group. The ID3 and ID4 groups had significantly greater weighted unifrac distance to the ID2 group compared to the ID2 group. In the visualized weighted unifrac beta diversity (Figure 3), different infection doses seemed clustered, and axis 1 score was comparatively high (>35%).

### 3.2. Phylum- and Family-Level Composition of the Cecal Microbial Communities

**Figure 4:**
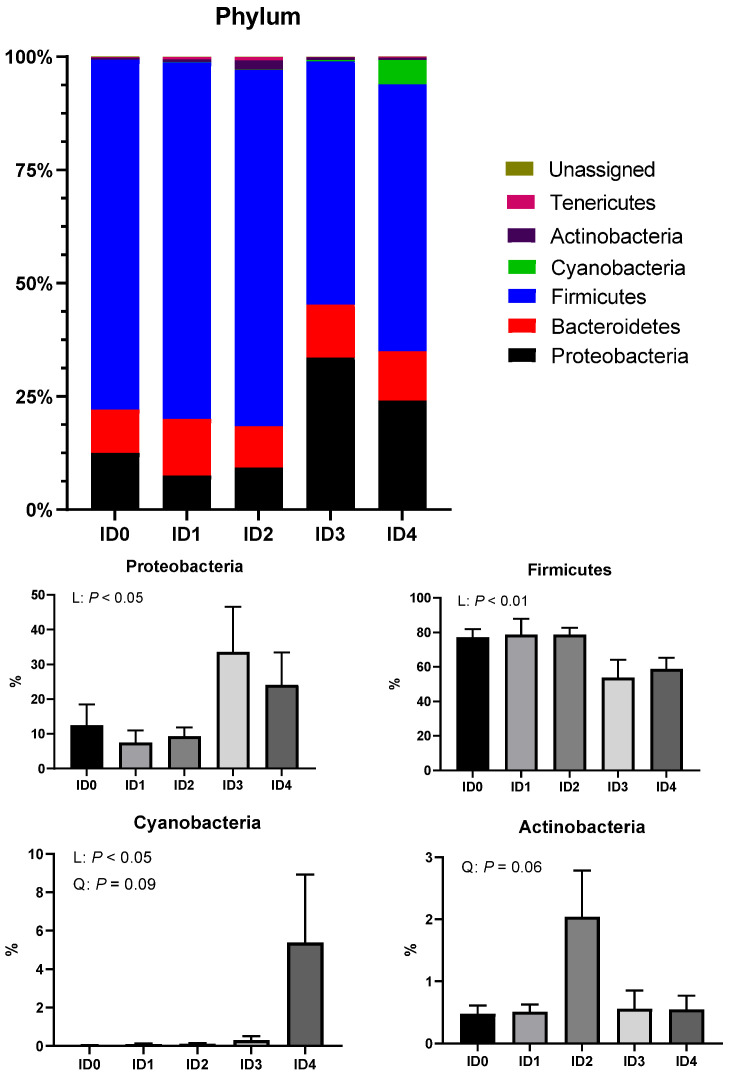
Effects of different infection doses of *Eimeria tenella* on phylum-level composition of the cecal microbial communities in broilers 6 days post-infection. Phylum-level composition was measured in the ID0 (infection dose 0; sham-challenged with phosphate-buffered saline); ID1 (infection dose 1; challenged with 6250 sporulated oocysts of *Eimeria tenella*); ID2 (infection dose 2; challenged with 12,500 sporulated oocysts of *E. tenella*); ID3 (infection dose 3; challenged with 25,000 sporulated oocysts of *E. tenella*); ID4 (infection dose 4; challenged with 50,000 sporulated oocysts of *E. tenella*) groups. Data were analyzed by using One-way ANOVA followed by the Tukey’s multiple comparison test. Orthogonal polynomial contrast analysis was used to assess linear (L) and quadratic (Q) effects of the infection doses, and *p* < 0.1 were presented.

The phylum level composition of cecal microbial communities is shown in Figure 4. The relative abundance of the phylum Proteobacteria was linearly increased by *Eimeria tenella* infection (*p* < 0.05). The relative abundance of the phylum Firmicutes was linearly reduced by *Eimeria tenella* infection (*p* < 0.01). *Eimeria tenella* infection linearly (*p* < 0.05) and quadratically (tendency; *p* = 0.09) increased the relative abundance of the phylum Cyanobacteria. The relative abundance of Actinobacteria tended to be quadratically increased by *Eimeria tenella* infection (*p* = 0.06).

**Figure 5:**
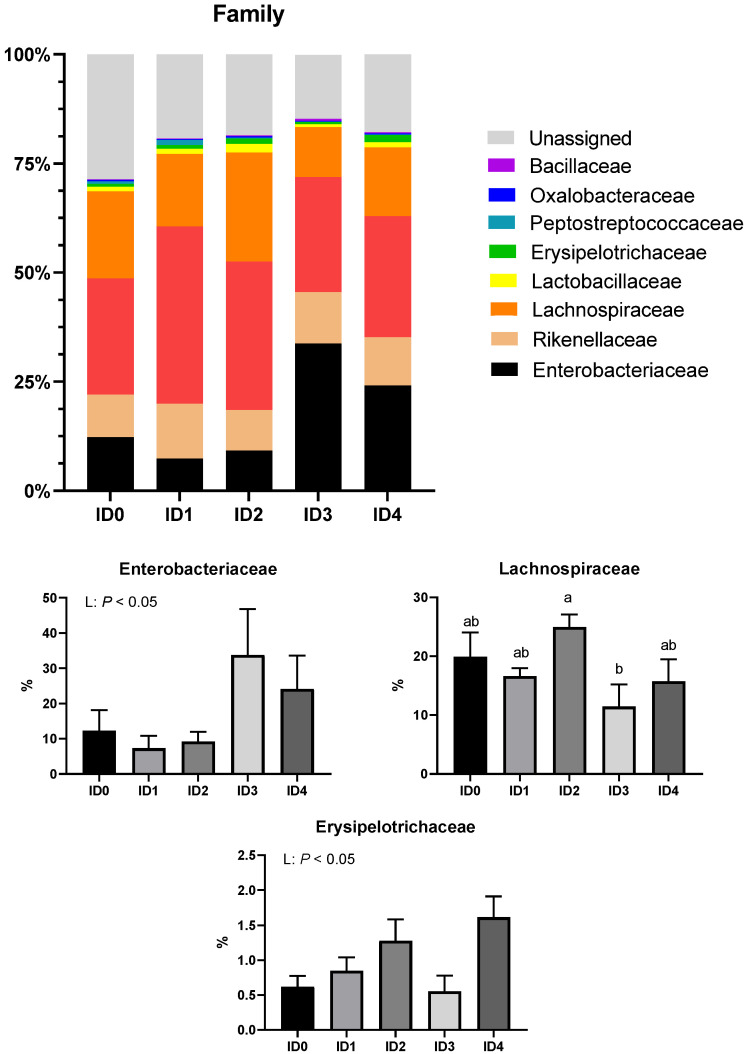
Effects of different infection doses of *Eimeria tenella* on family-level composition of the cecal microbial communities in broilers 6 days post-infection. Phylum-level composition was measured in the ID0 (infection dose 0; sham-challenged with phosphate-buffered saline); ID1 (infection dose 1; challenged with 6250 sporulated oocysts of *Eimeria tenella*); ID2 (infection dose 2; challenged with 12,500 sporulated oocysts of *E. tenella*); ID3 (infection dose 3; challenged with 25,000 sporulated oocysts of *E. tenella*); ID4 (infection dose 4; challenged with 50,000 sporulated oocysts of *E. tenella*) groups. Data were analyzed by using One-way ANOVA followed by the Tukey’s multiple comparison test and means with different letters (a and b) differ significantly (*p* < 0.05). Orthogonal polynomial contrast analysis was used to assess linear (L) and quadratic (Q) effects of the infection doses, and *p* < 0.1 were presented.

**Figure 6:**
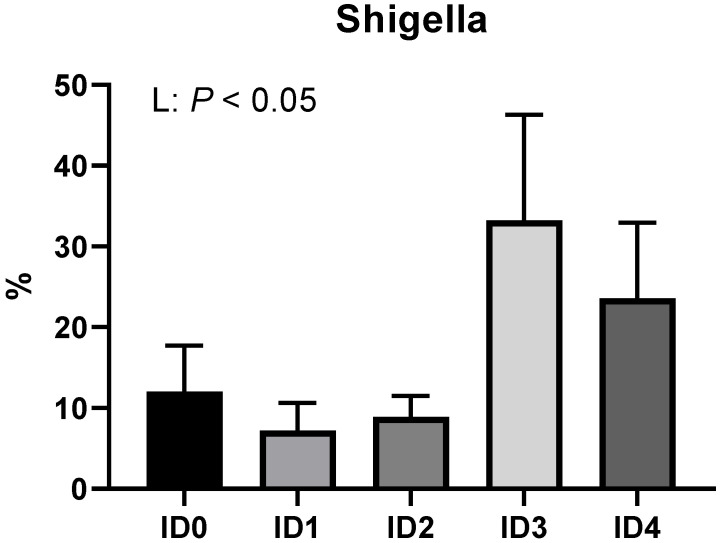
Effects of different infection doses of *Eimeria tenella* on relative abundance of genus *Shigella* in broilers 6 days post-infection. The relative abundance of genus *Shigella* was measured in the ID0 (infection dose 0; sham-challenged with phosphate-buffered saline); ID1 (infection dose 1; challenged with 6250 sporulated oocysts of *Eimeria tenella*); ID2 (infection dose 2; challenged with 12,500 sporulated oocysts of *E. tenella*); ID3 (infection dose 3; challenged with 25,000 sporulated oocysts of *E. tenella*); ID4 (infection dose 4; challenged with 50,000 sporulated oocysts of *E. tenella*) groups. Data were analyzed by using One-way ANOVA followed by the Tukey’s multiple comparison test. Orthogonal polynomial contrast analysis was used to assess linear (L) and quadratic (Q) effects of the infection doses, and *p* < 0.1 were presented.

Figure 5 presents the family level composition of cecal microbial communities. *Eimeria tenella* infection linearly increased the relative abundance of the family Enterobacteriaceae (*p* < 0.05). The ID2 group had a significantly higher relative abundance of the family Lachnospiraceae compared to the ID3 group. The relative abundance of Erysipelotrichaceae was linearly increased by *Eimeria tenella* (*p* < 0.05). *Eimeria tenella* infection linearly increased the relative abundance of the genus Shigella (*p* < 0.05; Figure 6).

### 3.3. Concentration of Protein and Secretory Immunoglobulin A (sIgA) in the Cecal Contents

**Figure 7:**
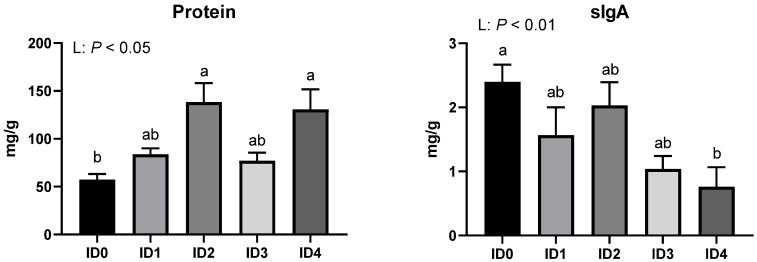
Effects of different infection doses of *Eimeria tenella* on concentration of protein and secretory immunoglobulin A (sIgA) in the cecal contents of broilers 6 days post-infection. Concentration of protein and secretory immunoglobulin A (sIgA) were measured in the ID0 (infection dose 0; sham-challenged with phosphate-buffered saline); ID1 (infection dose 1; challenged with 6250 sporulated oocysts of *Eimeria tenella*); ID2 (infection dose 2; challenged with 12,500 sporulated oocysts of *E. tenella*); ID3 (infection dose 3; challenged with 25,000 sporulated oocysts of *E. tenella*); ID4 (infection dose 4; challenged with 50,000 sporulated oocysts of *E. tenella*) groups. Data were analyzed by using One-way ANOVA followed by the Tukey’s multiple comparison test and means with different letters (a and b) differ significantly (*p* < 0.05). Orthogonal polynomial contrast analysis was used to assess linear (L) and quadratic (Q) effects of the infection doses, and *p* < 0.1 were presented.

The concentrations of proteins and sIgA in the cecal contents in broilers infected with *Eimeria tenella* were shown in Figure 7. Protein concentration in the cecal contents was linearly increased by *Eimeria tenella* infection (*p* < 0.05), and the ID2 and ID4 groups had significantly higher protein concentrations in the cecal content compared to the ID0 group. *Eimeria tenella* infection significantly reduced sIgA concentration in the cecal contents (*p* < 0.01), and the ID4 group had a significantly lower sIgA concentration compared to the ID0 group.

### 3.4. Goblet Cell Number per a Crypt and Goblet Cell Density in the Ceca

**Figure 8:**
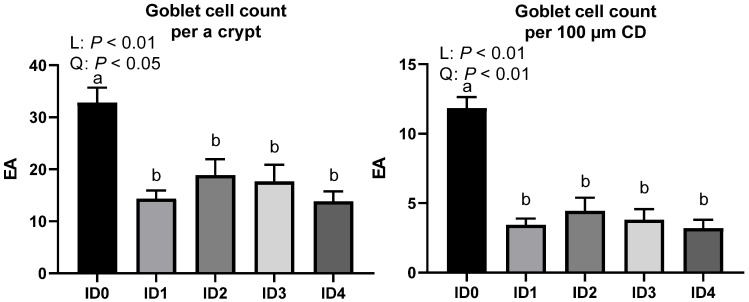
Effects of different infection doses of *Eimeria tenella* on goblet cell number per a crypt and goblet cell number per 100 μm crypt depth in the ceca of broilers 6 days post-infection. Goblet cell number and density were determined in the ID0 (infection dose 0; sham-challenged with phosphate-buffered saline); ID1 (infection dose 1; challenged with 6250 sporulated oocysts of *Eimeria tenella*); ID2 (infection dose 2; challenged with 12,500 sporulated oocysts of *E. tenella*); ID3 (infection dose 3; challenged with 25,000 sporulated oocysts of *E. tenella*); ID4 (infection dose 4; challenged with 50,000 sporulated oocysts of *E. tenella*) groups. Data were analyzed by using One-way ANOVA followed by the Tukey’s multiple comparison test and means with different letters (a and b) differ significantly (*p* < 0.05). Orthogonal polynomial contrast analysis was used to assess linear (L) and quadratic (Q) effects of the infection doses, and *p* < 0.1 were presented.

**Figure 9:**
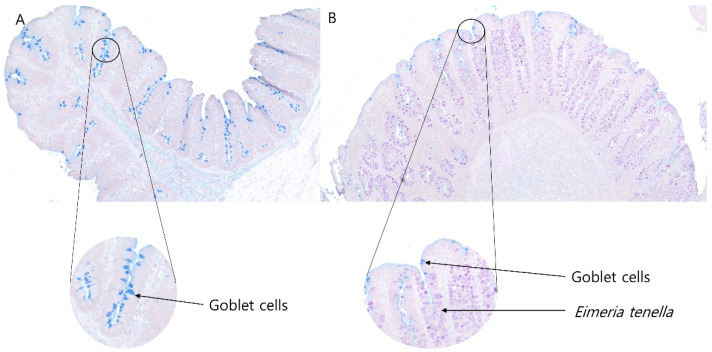
Goblet cells in the cecal crypt of broilers in healthy conditions (**A**) and of broilers infected with *Eimeria tenella* (**B**) on 6 days post-infection.

Goblet cell number per crypt and goblet cell number per 100 μm crypt depth in the ceca of broilers infected with *Eimeria tenella* are presented in Figure 8. Goblet cell count per a crypt was linearly (*p* < 0.01) and quadratically (*p* < 0.05) reduced, and the ID0 group had the highest goblet cell count per crypt (*p* < 0.05). Goblet cell count per 100 μm crypt was linearly (*p* < 0.01) and quadratically (*p* < 0.01) decreased, and the ID0 group had the highest goblet cell count per crypt (*p* < 0.05). There were obviously fewer goblet cells in the cecal crypts (Figure 9).

### 3.5. Relative mRNA Expression

**Figure 10:**
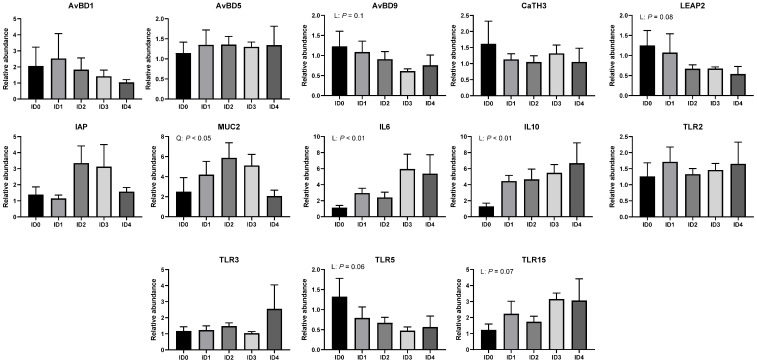
Effects of different infection doses of *Eimeria tenella* on relative mRNA expression of genes related to antimicrobial peptides, alkaline phosphatase, mucin gene, inflammatory cytokines, and toll-like receptors in the cecal tissue of broilers 6 days post-infection. Relative mRNA expression was determined in the ID0 (infection dose 0; Sham-challenged with phosphate-buffered saline); ID1 (infection dose 1; challenged with 6250 sporulated oocysts of *Eimeria tenella*); ID2 (infection dose 2; challenged with 12,500 sporulated oocysts of *E. tenella*); ID3 (infection dose 3; challenged with 25,000 sporulated oocysts of *E. tenella*); ID4 (infection dose 4; challenged with 50,000 sporulated oocysts of *E. tenella*) groups. Data were analyzed by using One-way ANOVA followed by the Tukey’s multiple comparison test. Orthogonal polynomial contrast analysis was used to assess linear (L) and quadratic (Q) effects of the infection doses, and *p* < 0.1 were presented.

Relative mRNA expression of genes related to antimicrobial peptides, mucin (MUC), interleukin (IL), and toll-like receptor (TLR) is shown in Figure 10. Relative mRNA expression of avian beta defensin 9 (AvBD9) tended to be linearly reduced by *Eimeria tenella* infection (*p* = 0.1). *Eimeria tenella* infection tended to linearly decrease mRNA expression of liver-expressed antimicrobial peptide 2 (LEAP2; *p* = 0.08). Relative mRNA expression of MUC2 was quadratically modulated by *Eimeria tenella* infection (*p* < 0.05). Relative mRNA expression of IL6 and IL10 was linearly increased by *Eimeria tenella* infection (*p* < 0.01). *Eimeria tenella* infection tended to linearly decrease mRNA expression of TLR5 (*p* = 0.06) and TLR15 (*p* = 0.07).

### 3.6. Alkaline Phosphatase Activities in the Cecal Tissue and Serum and Concentration of Serum Endotoxins in Broilers

**Figure 11:**
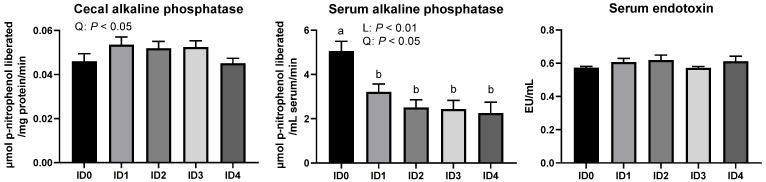
Effects of different infection doses of *Eimeria tenella* on alkaline phosphatase activities in the cecal tissue and serum and serum endotoxin concentration in broilers 6 days post-infection. Alkaline phosphatase activities in the cecal tissue and serum and serum endotoxin concentration determined in the ID0 (infection dose 0; sham-challenged with phosphate-buffered saline); ID1 (infection dose 1; challenged with 6250 sporulated oocysts of *Eimeria tenella*); ID2 (infection dose 2; challenged with 12,500 sporulated oocysts of *E. tenella*); ID3 (infection dose 3; challenged with 25,000 sporulated oocysts of *E. tenella*); ID4 (infection dose 4; challenged with 50,000 sporulated oocysts of *E. tenella*) groups. Data were analyzed by using One-way ANOVA followed by the Tukey’s multiple comparison test and means with different letters (a and b) differ significantly (*p* < 0.05). Orthogonal polynomial contrast analysis was used to assess linear (L) and quadratic (Q) effects of the infection doses, and *p* < 0.1 were presented.

Alkaline phosphatase activities in the cecal tissue and serum and concentration of serum endotoxins in broilers are shown in Figure 11. Alkaline phosphatase activities in the cecal tissue were quadratically enhanced in broilers infected with *Eimeria tenella* (*p* < 0.05). *Eimeria tenella* infection linearly (*p* < 0.01) and quadratically (*p* < 0.05) decreased activities of serum alkaline phosphatase, and the ID0 group had the higher serum alkaline phosphatase activities compared to the ID1, 2, 3, 4 groups (*p* < 0.05). No differences were observed in the concentration of serum endotoxins among all groups (*p* > 0.1).

## 4. Discussion

The objective of the current study was to investigate the effects of *Eimeria tenella* infection on the cecal microbiome, protein concentration of cecal content, cecal mucosal immunity, and serum endotoxin levels of broilers. In the current study, *Eimeria tenella* infection decreased alpha diversity parameters including shannon entropy (richness and evenness) and pielou’s evenness (evenness) in broilers, which indicates that cecal microbial communities were imbalanced due to *Eimeria tenella* infection [27]. In agreement, a previous study by Abbas et al. [28] reported that *Eimeria tenella* infection reduced shannon entropy in the cecal microbial communities in broilers. Lower alpha diversity may suggest unhealthy and undeveloped microbial communities [9]. Moreover, statistical differences were observed in the alpha diversity parameters including shannon entropy and Pielou’s evenness between the ID2 and ID3 groups due to *Eimeria tenella* infection. This indicates that different infection doses of *Eimeria tenella* can induce a modulation in the alpha diversity parameters in the cecal microbial communities among *Eimeria tenella* infected groups. Beta diversity analyses showed that there were differences in the bacterial sequences between the non-infected and infected groups and among the infected groups in the current study. In agreement with this, Macdonald et al. [11] reported that *Eimeria tenella* infection induced differences in beta diversity parameters in broilers.

In the present study, the relative abundance of the phylum Proteobacteria was linearly increased by *Eimeria tenella* infection. The phylum Proteobacteria is a major phylum of Gram-negative bacteria and includes diverse pathogens including *Salmonella* spp. and *Escherichia coli* [29]. In healthy broiler chickens, the relative abundance of the phylum Proteobacteria accounts for less than 10% in the cecal microbial communities [30], and the relative abundance of the phylum Proteobacteria in the cecal microbial communities of uninfected group in the current study was around 10%. However, the ID3 and ID4 groups had around 20 to 30% of the relative abundance of the phylum Proteobacteria. Along with the increase in the relative abundance of the phylum Proteobacteria, the relative abundance of the family Enterobacteriaceae, which contains a variety kind of pathogens such as Klebsiella, Salmonella, Escherichia, Enterobacter, Yersinia, Proteus, Shigella, and Serratia [31], was increased in broilers infected with *Eimeria tenella*. The relative abundance of the phyla Cyanobacteria and Actinobacteria were linearly and quadratically increased, respectively, due to *Eimeria tenella* infection in the current study. The abundance of the phyla Cyanobacteria and Actinobacteria are nearly absent in the ceca of healthy broilers [30]. The phylum Cyanobacteria is a common pathogen found in water sources for broilers [32], and Zhang et al. [33] reported that the phylum Actinobacteria potentially can be harmful in broilers. The phyla Cyanobacteria and Actinobacteria cannot form their own microbial community in the ceca of healthy broilers, but compromised mucosal immunity due to *Eimeria tenella* infection may have facilitated their colonization in the ceca of broilers. *Eimeria tenella* infection increased the relative abundance of the phyla Proteobacteria, Cyanobacteria, and Actinobacteria and the family Enterobacteriaceae, which includes many pathogens, and this may have led to malfunctions in the gastrointestinal tract of broiler chickens.

The relative abundance of the phylum Firmicutes was linearly reduced by *Eimeria tenella* infection in the current study. Firmicutes are the main phylum that produce VFA, which are important energy sources for the host [34]. In our previous study [6], *Eimeria tenella* infection linearly decreased cecal VFA concentration, which led to a decrease in feed efficiency in broiler chickens. Hence, *Eimeria tenella* infection reduced the relative abundance of the phylum Firmicutes, leading to a decrease in VFA production and feed efficiency in broilers. The relative abundance of the family Erysipelotrichaceae was linearly increased by *Eimeria tenella*, and it is reported that the family Erysipelotrichaceae was negatively correlated with body fat, intestinal health, and butyrate production [35]. In the current study, *Eimeria tenella* infection modulated microbes related to VFA production in broilers.

Microbiota of the gastrointestinal tract can be modulated by nutrient composition of digesta content [36]. The phylum Proteobacteria, which cannot use complex carbohydrates as an energy source, mainly use diet (soybean meal)- or host (mucus and cellular debris) originated protein and amino acid sources [37]. Because most of the proteins are digested in the upper gastrointestinal tract of chickens, the abundance of the phylum Proteobacteria is relatively lower in the lower gastrointestinal tract compared to the upper gastrointestinal tract. In the present study, the total protein concentration of cecal content, measured by BCA method [36], was linearly increased by *Eimeria tenella* infection. In the ceca of chickens, host-derived proteins mainly include mucus, immunoglobulins, cell debris, enzymes, etc. However, our present study showed that concentrations of mucus and immunoglobulins in the cecal content were reduced by *Eimeria tenella* in broilers, which potentially indicates that increased protein concentration would be mainly due to cell debris from the enterocytes. In the current study, *Eimeria tenella* invaded crypts and took spaces in the crypts (Figure 9), which may have produced host cell debris. Moreover, during the asexual and sexual replications of *Eimeria tenella*, sprozoite, merozoite, microgamete, and oocysts go in and out the enterocytes, which may also shed host cell debris to the cecal content. Witlock et al. [38] visualized sloughed cell debris from cecal tissue due to *Eimeria tenella* infection in broilers by using scanning electron microscopy. Moreover, cecal crypts were thickened by *Eimeria tenella* infection potentially by decreasing cell apoptosis in the ceca [39], and this can aggravate the increase in the concentration of host cell debris in the cecal content by increasing the number of the enterocytes. Increased protein concentration of cecal contents by shedding host cell debris would be one of the factors to increase the abundance of the phylum Proteobacteria in broilers infected with *Eimeria tenella.* In addition to alteration in microbiota, increased protein concentration in the cecal content may increase protein fermentation, which produces harmful metabolites, such as ammonia, biogenic amines, hydrogen sulfides, and nitric oxide [40].

Mucosal immunity is closely associated with microbial communities in chickens [41]. Secretory immunoglobulin A (sIgA) is the main predominant antibody that is secreted to the intestinal lumen along with IgY and IgM in chickens [42]. The sIgA exhibits antigen-specific immunity and plays an important role in controlling pathogens and toxins [43]. Moreover, commensal bacteria can be protected by coating procedure of sIgA [44]. Production and secretion of mucosal IgA can be increased with the upregulation of IL6 and IL10 from T helper cells [45]. In the current study, *Eimeria tenella* infection linearly decreased sIgA concentration in the cecal content, but linearly increased mRNA expression IL6 and IL10 in the cecal tissue. Potentially, the mucosal immune system focused on increasing *Eimeria tenella*-specific immunoglobulin A in the inner mucus layer as a defensive system against *Eimeria tenella* but could not maintain the sIgA level in the cecal content to control pathogenic bacteria (e.g., Proteobacteria) based on increased mRNA expression of IL6 and IL10 and decreased sIgA in the luminal content in the current study. This is because *Eimeria tenella*-specific immunoglobulin A mainly presents in the cecal wall (inner mucus layer) [46], but sIgA concentration in the cecal content was measured in the current study. Consistently, Tian et al. [47] reported that the concentration of sIgA was reduced in the ceca of broilers infected with *Eimeria tenella*. However, a study by Bun et al. [48] reported that *Eimeria tenella* infection increased sIgA concentration in the cecal content. The differences in immune response to *Eimeria tenella* would be originated from different *Eimeria tenella* strain, animals (strain and age), and experimental conditions.

Antimicrobial peptides, which are expressed by Paneth cells and enterocytes, also play crucial roles in controlling microbiota and protecting from the pathogenic bacteria in the gastrointestinal tract of chickens [49]. Antimicrobial peptides belong to the innate immune system and provide a quick immune response against a broad range of pathogens [50]. In the current study, *Eimeria tenella* infection linearly downregulated antimicrobial peptide genes including AvBD9 and LEAP2 in the cecal tissue. Each AvBD provides protection against specific pathogenic bacteria [51]. Yacoub et al. [52] reported that AvBD9 showed bactericidal effects against *Staphylococcus aureus* and *Shigella sonni* via modulating the membrane of the pathogenic bacteria. Our current study also showed that *Eimeria tenella* infection linearly increased the relative abundance of the genus Shigella in the cecal contents of broilers. Potentially reduced mRNA expression of AvBD9 due to *Eimeria tenella* infection would be closely associated with the increase in the relative abundance of the genus Shigella. A previous study by Su et al. [53] also demonstrated that mRNA expression of LEAP2 was downregulated in *Eimeria* spp.-infected broilers. Modulated mRNA expression of antimicrobial peptides would be potentially associated with an altered microbiome in the cecal contents of broilers infected with *Eimeria tenella.*

Mucus, which is synthesized and secreted by goblet cells, plays an important role in protecting epithelial cell lining by providing physical barriers and provide habitats and food for microbiota [54]. In the current study, *Eimeria tenella* infection reduced goblet cell number per crypt and goblet cell density in the cecal tissue of broilers. Similarly, Qasem et al. [8] showed that *Eimeria tenella* infection reduced the number of goblet cells in the ceca, and the potential reason would be that *Eimeria* spp. destroys stem cells that can be differentiated to goblet cells. Poor production of mucus can cause physical contacts between pathogens and host cells, which can induce inflammation in chickens [55] and decrease the abundance of commensal bacteria by reducing their food and habitats [56]. The commensal bacteria (e.g., Firmicutes and Bacteroidetes) in the large intestine have an ability to use mucin glycans (e.g., polysaccharides) by using their glycan-degrading enzymes [57]. Potentially, reduced goblet cells in the current study might have reduced mucin production, which caused a decrease in the relative abundance of the phylum Firmicutes due to lack of their food and limited habitats. However, mRNA expression of MUC2, the major MUC in the ceca of chickens [58], quadratically increased in the current study, which would be a defensive mechanism against reduced mucus production due to a decreased goblet cell number.

**Figure 12:**
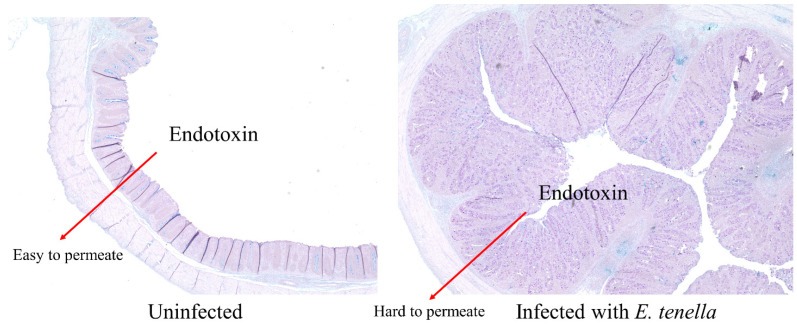
Pictures of thickened mucus layer in the ceca of broilers infected with *Eimeria tenella* and their proposed effects on the permeation of endotoxin.

The endotoxins (lipopolysaccharides; LPS), which are mainly produced from the phylum Proteobacteria and are important cell wall components of the phylum Proteobacteria, induce sepsis (strong inflammatory response due to blood LPS) and can cause mortality in broilers [59]. Mutlu et al. [60] showed that the abundance of the phylum Proteobacteria in the colon is positively correlated with serum endotoxin level. *Eimeria tenella* have been known to induce severe mortality compared to other *Eimeria* spp. in chickens [61]. We hypothesized that an increase in the relative abundance of Proteobacteria in the ceca may increase serum endotoxin level and mortality in broilers infected with *Eimeria tenella*. However, in the current study, no differences were observed in the serum LPS concentration and mortality, while the relative abundance of the phylum Proteobacteria was increased in broilers infected with *Eimeria tenella*. The potential reason would be that LPS produced in the luminal side were less likely to permeate across the thickened epithelium due to *Eimeria tenella* infection (Figure 12). More studies are required to investigate whether a severe *Eimeria tenella* infection model can increase the serum endotoxin level and mortality in broilers.

Alkaline phosphatase in the intestine has an important role in modulating microbiota and detoxifying LPS via ATP and LPS dephosphorization, respectively [62]. The intestinal alkaline phosphatase can induce the growth of the commensal microbe via the dephosphorization of ATP, which inhibits Gram-positive bacteria in the intestinal lumen [63]. While alkaline phosphatase can detoxify free LPS, alkaline phosphatase cannot detoxify LPS in the intact bacterial cell wall [64], which implies that intestinal alkaline phosphatase production is directly associated with the relative abundance of the Gram-negative bacteria. In the current study, *Eimeria tenella* infection quadratically increased the activities of intestinal alkaline phosphatase in the cecal tissues. Potentially, it was a defensive mechanism against high LPS production by the increased growth of the phylum Proteobacteria in the ceca. However, alkaline phosphatase activities in the serum, which play an important role in detoxifying LPS in the blood, were linearly decreased by *Eimeria tenella* infection in broilers. This could be explained by the possibility that reduced VFA production in the ceca by *Eimeria tenella* infection may have resulted in decreased serum alkaline phosphatase activities because VFA have a function to activate alkaline phosphatase [65]. The reduced activities of alkaline phosphatase in the serum suggest that *Eimeria tenella* infection can make broilers vulnerable to sepsis.

The TLRs are important components of the innate immunity by distinguishing pathogen-associated molecular patterns in the microbiota and play critical roles in stimulating the immune system and regulating immune cells [66]. In the current study, *Eimeria tenella* infection linearly reduced TLR5 and increased TLR15 in broilers. Each TLR has a unique affinity to different ligands, and the specific ligands of TLR5 and TLR15 are flagellin and bacterial protease, respectively [67]. In contrast, Zhang et al. [68] reported that *Eimeria tenella* infection upregulated TLRs including TLR4, TLR5, TLR7, and TLR21 in broilers. The differences were derived from different strains, inoculum doses, birds, and experiment conditions. *Eimeria tenella* infection was the main factor to alter immunity because *Eimeria tenella* directly exhibited physical damage to the cecal tissue, affecting the microbiome in the intestine of broilers [12]. Moreover, an altered microbiome due to *Eimeria tenella* infection also affects the host immune system. The TLRs may have been affected by increased the phylum Proteobacteria and the family Enterobacteriaceae, and this could also possibly further affect the immune system of broilers infected with *Eimeria tenella.*

In the current study, increased *Eimeria tenella* infection doses did not negatively affect the mRNA expression of MUC2 and activities of serum alkaline phosphatase with a linear trend, and the ID2 group showed numerically out of trend results compared to the ID1 and ID3 groups in the relative abundance of the phylum Actinobacteria and the family Lachnospiraceae and in the concentration of protein and sIgA in the cecal content. Potentially, this may have been because (1) broiler chickens activated the mucosal immune system against low or specific doses of pathogens as defensive reactions (e.g., hormesis) [69] and (2) doses of the ID2 group (12,500) would be more pathogenic compared to the ID3 (25,000) and ID4 (50,000) groups because the effects of high Eimeria infection doses can be limited due to crowding effects of *Eimeria* spp. [70]. This suggests that higher infection doses of *Eimeria tenella* do not always guarantee more severe negative effects on the gut microbiome and mucosal immunities in broilers.

## 5. Conclusions

**Figure 13:**
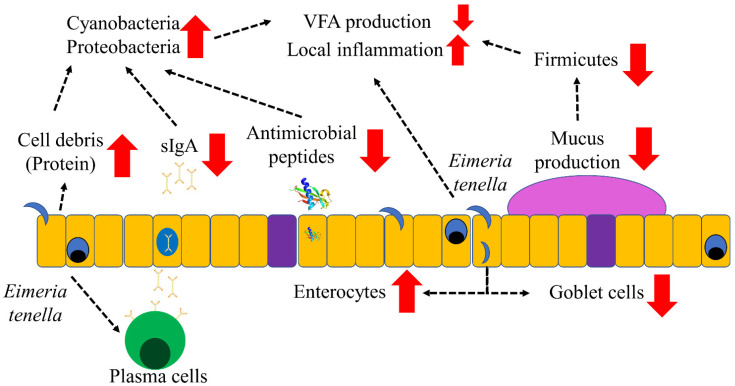
Summary of the interactions of microbiota and mucosal immunity in the ceca of broiler chickens infected with *Eimeria tenella.* Infection of *Eimeria tenella* increased protein concentrations potentially by increasing cell debris in the cecal contents. *Eimeria tenella* infection reduced concentrations of secretory immunoglobulin A (sIgA) and antimicrobial peptides. These factors would have increased the relative abundance of the phylum Proteobacteria and Cyanobacteria, considered pathogenic bacteria. *Eimeria tenella* infection reduced goblet cell density, which would have reduced mucus production in the ceca. This would have resulted in reducing the relative abundance of the Firmicutes, which use mucus for colonization and have important roles in degrading fiber and producing volatile fatty acids in the ceca. *Eimeria tenella* infection with negatively modulated microbiota would have induced local inflammation in the ceca of broiler chickens.

*Eimeria tenella* infection negatively affected cecal microbiota toward increasing pathogenic bacteria and decreasing microbial VFA production potentially by impairing both the adaptive and innate immune system and by increasing the protein concentration in the cecal contents of broilers (Figure 13). Hence, this study suggests that the supplementation of immunostimulatory agents (e.g., plant extract, probiotics, VFA, etc.) could potentially be used to maintain healthy microbiota in broilers infected with *Eimeria tenella*.

## Figures and Tables

**Table 1:** Composition of the experimental diet (g/kg, as fed basis).

Ingredients	D 14 to 20
Corn	700.8
Soybean meal (480 g crude protein/kg)	241.73
Soybean oil	15.84
Defluorinated phosphate	13.99
Sand	7.00
Limestone	6.11
Titanium dioxide	3.00
DL-Methionine 99%	2.86
L-Lysine HCl 78%	2.80
Vitamin Premix ^1^	2.50
Sodium chloride	1.79
L-threonine	0.77
Mineral Premix ^2^	0.80
Total	1000
Calculated energy and nutrient value, %	
Metabolizable energy, kcal/kg	3100
Crude protein	18.375
SID ^3^ Methionine	0.552
SID Total sulfur amino acids	0.8
SID Lysine	1.02
SID Threonine	0.66
Total calcium	0.76
Available phosphate	0.38

^1^ provided in mg/100 g diet: thiamine-HCl, 1.5; riboflavin 1.5; nicotinic acid amide 15; folic acid 7.5; pyridoxine-HCl, 1.2; d-biotin 3; vitamin B-12 (source concentration, 0.1%) 2; d-calcium pantothenate 4; menadione sodium bisulfite, 1.98; α-tocopherol acetate (source 500,000 IU/g), 22.8; cholecalciferol (source 5,000,000 IU/g) 0.09; retinyl palmitate (source 500,000 IU/g), 2.8; ethoxyquin, 13.34; I-inositol, 2.5; dextrose, 762.2. ^2^ provided in g/100 g diet: Ca(H_2_PO_4_)_2_ · H_2_O, 3.62; CaCO_3_, 1.48; KH_2_PO_4_, 1.00; Na_2_SeO_4_, 0.0002; MnSO_4_ · H_2_O, 0.035; FeSO_4_ · 7H_2_O, 0.05; MgSO_4_ · 7H_2_O, 0.62; KIO_3_, 0.001; NaCl, 0.60; CuSO_4_ · 5H_2_O, 0.008; ZnCO_3_, 0.015; CoCl_2_ · 6H_2_O, 0.00032; NaMoO_4_ · 2H_2_O, 0.0011; KCl, 0.10; dextrose, 0.40. ^3^ SID: standard ileal digestible amino acid.

**Table 2:** Primers for analyzing the relative mRNA abundance of antimicrobial peptides, alkaline phosphatase, mucin gene, inflammatory cytokines, and toll-like receptors ^1^.

Target	Primers	Size of PCR Product
GAPDH	5′-GCTAAGGCTGTGGGGAAAGT-3′	161
	5′-TCAGCAGCAGCCTTCACTAC-3′	
Beta actin	5′-CAACACAGTGCTGTCTGGTGGTA-3′	205
	5′-ATCGTACTCCTGCTTGCTGATCC-3′	
AvBD1	5′-GGATCGTGTACCTGCTCCTC-3′	113
	5′-TGCACAGAAGCCACTCTTTC-3′	
AvBD5	5′-CTCTTTGCTGTCCTCCTCCT-3′	118
	5′-CTGGAGGACATGACTTGTGG-3′	
AvBD9	5′-GCTGACACCTTAGCATGCAG-3′	113
	5′-CATTTGCAGCATTTCAGCTT-3′	
CaTH3	5′-GCTGTGGACTCCTACAACCA-3′	124
	5′-CCATGATGGTGAAGTTGAGG-3′	
LEAP2	5′-TATTCTTCTCGCTGCTGCTC-3′	123
	5′-AGGCTCCAACAGGTCTCAGT-3′	
Alkaline phosphatase	5′-CTTCCTCGGAGATGGATTTG-3′	123
	5′-AGAGCCACATAGGGGAAAGA-3′	
MUC2	5′-ATGCGATGTTAACACAGGACTC-3′	110
	5′-GTGGAGCACAGCAGACTTTG-3′	
IL6	5′-ATAAATCCCGATGAAGTGG-3′	146
	5′-CTCACGGTCTTCTCCATAAA-3′	
IL10	5′-CTGTCACCGCTTCTTCACC-3′	85
	5′-CCCGTTCTCATCCATCTTCT-3′	
TLR2	5′-CGGTGGAAAGGGAGAAAG-3′	103
	5′-CTTGCCACATCAGCTTCATT-3′	
TLR3	5′-GGCTAAACGACACTCAAGCA-3′	113
	5′-CTTGCAGGCTGAGGTATCAA-3′	
TLR5	5′-CGTTAGTGAGAATGGCTGGA-3′	106
	5′-TGAGCCCATTGTATGAGAGC-3′	
TLR15	5′-ATTGAACCTGGTGCATTTGA-3′	102
	5′-TTTCAGGTGAGGTGCAAGAC-3′	

^1^ GAPDH, glyceraldehyde 3-phosphate dehydrogenase; AvBD, avian beta defensin; CaTH3, Cathelicidin 3; LEAP2, liver-expressed antimicrobial peptide 2; MUC2, mucin 2; IL, interleukin; TLR, toll-like receptor.

## Data Availability

Not applicable.
